# MRSA Transmission between Cows and Humans

**DOI:** 10.3201/eid1304.060833

**Published:** 2007-04

**Authors:** Éva Juhász-Kaszanyitzky, Szilárd Jánosi, Pál Somogyi, Ádám Dán, Linda vanderGraaf van Bloois, Engeline van Duijkeren, Jaap A. Wagenaar

**Affiliations:** * Central Veterinary Institute, Budapest, Hungary; † Animal Sciences Group, Lelystad, the Netherlands; ‡ Utrecht University, Utrecht, the Netherlands

**Keywords:** Methicillin-resistant *Staphylococcus aureus*, cows, mastitis, zoonosis, phage types, PFGE, SCC*mec* type, *spa*-typing, MLST, dispatch, Suggested citation for this article: Juhász-Kaszanyitzky E, Jánosi S, Somogyi P, Dán A, van der Graaf-van Bloois L, van Duijkeren E, et al. MRSA transmission between cows and humans. Emerg Infect Dis [serial on the Internet]. 2007 Apr [*date cited*]. Available from http://www.cdc.gov/eid/content/13/4/630.htm

## Abstract

We isolated methicillin-resistant *Staphylococcus aureus* (MRSA) from cows with subclinical mastitis and from a person who worked with these animals. The bovine and human strains were indistinguishable by phenotyping and genotyping methods and were of a low frequency *spa* type. To our knowledge, this finding indicates the first documented case of direct transmission of MRSA between cows and humans.

Since the introduction of β-lactamase–stable antimicrobial drugs into clinical use, methicillin-resistant *Staphylococcus aureus* (MRSA) strains have emerged worldwide as important nosocomial pathogens; their prevalence in the community is increasing substantially. Although *S. aureus* is known to be one of the most common causes of bovine mastitis and other severe animal diseases such as septicemia and wound, bone, and joint infections, MRSA strains have been rarely isolated from animals. MRSA strains have been isolated from cows with mastitis, horses and dogs with lesions, and dogs and cats that were carriers (*1*). Transmission of MRSA between humans and animals (e.g., dogs, horses, pigs) has been reported (*2**–**4*), but transmission between cows and humans has not, to our knowledge. We describe a first putative case of transmission of MRSA between cows and a person.

From January 2002 through December 2004, 595 milk samples were collected from cows with subclinical mastitis on a farm in Hungary and sent for bacteriologic analysis to the Bacteriological Department of the Hungarian Central Veterinary Institute. Samples were streaked onto a Columbia agar plate (Merck, Darmstadt, Germany) containing 5% sheep blood and 0.01% esculin and a Baird-Parker (BP) agar plate (Oxoid Ltd., Basingstoke, England). After incubation at 37°C for 24 h, the colonies were tentatively identified according to morphologic features, pigment production, Gram stain results, catalase test results, type of hemolysis, and characteristic growth on BP agar. The isolates initially characterized as staphylococci were tested for coagulase production (in tubes) and with Slidex Staph Plus test (bioMérieux, Marcy l'Etoile, France) to confirm their identification as *S. aureus*. From this farm, 375 *S. aureus* strains were isolated. The strains were tested for antimicrobial drug susceptibility, production of β-lactamases, and presence of *mec*A by PCR (*5*). The first MRSA strain was isolated in spring 2002; during the next 15 months, 26 additional MRSA strains were isolated from this dairy herd.

In December 2002, tonsil swabs were collected once from 12 workers on this farm who were in close contact with the cows (veterinarian, milkmen, and attendants) and who gave informed consent (the study was approved by the Ethical Committee of the National Center for Epidemiology, Budapest, Hungary). Culturing and identification of *S. aureus* were carried out by the above-described method. *S. aureus* was isolated from 3 samples. One of these isolates was resistant to methicillin by disk diffusion and E-test, and the presence of the *mec*A gene was confirmed by PCR.

All 28 MRSA strains (27 bovine and 1 human) produced PBP2a, according to latex agglutination test (Oxoid Ltd.). Eight of 27 randomly chosen bovine strains and the human strain containing the *mec*A gene, as well as 4 bovine and 2 human *mec*A–negative isolates, were phage typed with MRSA phages (*6*) at the Institute of National Public Health and Medical Officers Service in Hajdú-Bihar County. The phages were used in 2 concentrations: routine test dilution (RTD), and 100× RTD. None of the MRSA or methicillin-susceptible *S. aureus* (MSSA) strains were lysed by phages in RTD. All MRSA strains showed a similar lysis pattern with the 100× RTD MRSA phages (Table).

**Table:** Susceptibility of human and animal *Staphylococcus aureus* strains to phages and the presence of *mec*A by PCR*

Strain no.†	Reference no.	Identification of samples	*mec*A	MRSA 100× RTD‡^*^
1	13535	795 LF	+	616/617/623/626/630
2	16480	588 RF	+	616/617/623/626/630
3	24069/2	490 RF	+	616/617/623/626/630
4	24069/4	723 RH	+	616/617/623/626/630
5	24069/13	1572 LF	+	616/617/623/626/630
6	30195	632 RF	+	616/617/623/626/630
7	23457	1379 LH	+	616/617/623/626/630
	29509†	540 RH	+	616/617/623/626/630
8	Human/3		+	616/617/623/626/630
9	24069/9	381 LF	–	
10	24069/10	429 LH	–	
11	24069/11	519 LH	–	
12	24069/15	2551 RH	–	
13	Human/4		–	
14	Human/2		–	

*Strains isolated from cows with subclinical mastitis, Hungary, January 2002–December 2004. †See Figure for DNA analysis. Strain no. 29509 was not included in the DNA analysis; thus, no strain no. was assigned. ‡Reaction pattern with 100× routine test dilution (RTD) methicillin-resistant *Staphyloccocus aureus* phages (*7*). +, positive in *mec*A PCR; –, negative in *mec*A PCR.

Susceptibilities to 7 antimicrobial agents were assessed by a disk diffusion method that used the Clinical and Laboratory Standards Institute (CLSI; formerly National Committee for Clinical Laboratory Standards) breakpoints for 7 *mec*A–positive bovine strains and the human *mec*A–positive strain. All tested strains showed the same susceptibility pattern (resistant to ampicillin, cephalexin, tetracycline, and erythromycin and susceptible to enrofloxacin, gentamicin, and trimethoprim/sulfamethoxazole). Pulsed-field gel electrophoresis was performed on the 7 *mec*A–positive bovine strains, 1 human *mec*A–positive strain, 4 bovine MSSA strains, and 2 human MSSA strains, as described by McDougal et al. (*7*). The patterns of the *Sma*I–digested DNA of the strains are presented in the Figure. All bovine *mec*A–positive strains and the human *mec*A–positive strain (human 3) showed indistinguishable *Sma*I patterns. All *mec*A–negative isolates showed a pulsed-field gel electrophoresis pattern different from that of the MRSA strains.

**Figure:**
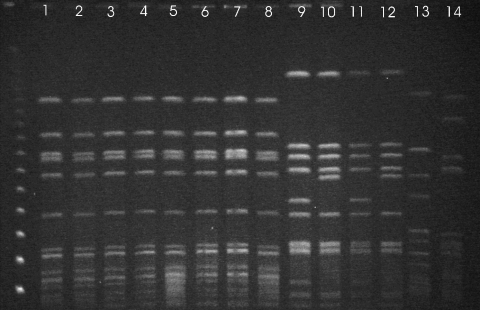
Pulsed-field gel electrophoresis patterns of *Sma*I-digested DNA of bovine and human strains of *Staphyloccocus aureus* isolated from cows with subclinical mastitis, Hungary, January 2002–December 2004.

For further identification, 1 human and 4 bovine MRSA isolates (29509, 24069/2, 24069/4, 30195; Table) underwent *spa* typing (*8*). All these isolates showed *spa*-type t127. Because the strains were indistinguishable by all methods, multilocus sequence typing (MLST) (*9*) and typing of the staphylococcal cassette chromosome (SSC*mec*) (*10*) were performed on just 1 strain (29509). This strain showed MLST sequence type (ST) 1 and SCC*mec* type IVa. The strain did not carry the Panton-Valentine leukocidin (PVL) toxin genes *lukF–lukS* as tested by PCR (*11*).

In this study, both phenotyping and genotyping showed that the MRSA isolates of bovine origin and the single human isolate were indistinguishable. The database of the European network for *spa* typing (www.seqnet.org) shows that MLST ST 1/*spa*-type t127 has a relative global frequency of 0.7%; according to this database, this type has not previously been reported in Hungary. A study of 135 human MRSA isolates collected in Hungary during 2001–2004 showed human epidemic clones of types other than ST 1 and *spa*-type t127 (*12*). We conclude that the bovine and human MRSA strains described in our study are epidemiologically related, which indicates transmission from either cow to human or human to cow. This strain is negative for the PVL genes, which differentiates it from community-associated MRSA ST 1, which is positive for PVL genes (*11*).

Several cows had positive test results for MRSA, which indicated that MRSA was spread within the farm. On the farm, cases of clinical mastitis were treated with intramammary infusions containing penicillin, aminopenicillins, or cephalosporins. Each cow also received drying-off therapy with cloxacillin or cephalosporins. The use of antimicrobial drugs may have contributed to the emergence of MRSA in this dairy farm.

MSSA strains with ST 1 and *spa*-type t127 have been reported from human sources (*13*). MSSA strains may be induced to pick up the *mec*A gene from coagulase-negative staphylococci. Alternatively, mastitis may be caused by human MRSA strains or bovine MRSA strains already present in small numbers and selected for by the frequent use of long-acting antimicrobial preparations, especially β-lactams. *S. aureus* usually shows limited host specificity, and transfer between different host species may occur (*14*). The transmission of milk-associated *S. aureus* strains between cows and humans was suggested by Lee (*15*), whose study showed MRSA in milk samples with comparable antibiotypes as those in humans, but the transfer to humans was not proven. The risk for spread of MRSA from bovine sources into the human population is low. Generally, persons are not at risk as long as raw milk is not consumed. However, persons in close contact with MRSA-infected cattle, including veterinarians, farmers, milkers, and persons working at slaughterhouses, may become colonized from the bovine source.

We conclude that several cases of subclinical mastitis in cows on a farm in Hungary were caused by MRSA and that these strains were indistinguishable from MRSA isolated from a carrier working in close contact with the cows. This suggests the transmission of these isolates between humans and cows, although the direction of transfer (cow to human or human to cow) could not be proven.
